# Quantitative Microbial Risk Assessment in Occupational Settings Applied to the Airborne Human Adenovirus Infection

**DOI:** 10.3390/ijerph13070733

**Published:** 2016-07-20

**Authors:** Annalaura Carducci, Gabriele Donzelli, Lorenzo Cioni, Marco Verani

**Affiliations:** 1 Laboratory of Hygiene and Environmental Virology, Department of Biology, University of Pisa, Via S. Zeno 35/39, Pisa 56127, Italy; annalaura.carducci@unipi.it (A.C.); marco.verani@unipi.it (M.V.); 2 Scuola Normale Superiore, Piazza dei Cavalieri 7, Pisa 56126, Italy; lcioni@di.unipi.it

**Keywords:** quantitative microbial risk assessment, airborne infectious, human adenovirus (HAdV), occupational exposure

## Abstract

Quantitative Microbial Risk Assessment (QMRA) methodology, which has already been applied to drinking water and food safety, may also be applied to risk assessment and management at the workplace. The present study developed a preliminary QMRA model to assess microbial risk that is associated with inhaling bioaerosols that are contaminated with human adenovirus (HAdV). This model has been applied to air contamination data from different occupational settings, including wastewater systems, solid waste landfills, and toilets in healthcare settings and offices, with different exposure times. Virological monitoring showed the presence of HAdVs in all the evaluated settings, thus confirming that HAdV is widespread, but with different average concentrations of the virus. The QMRA results, based on these concentrations, showed that toilets had the highest probability of viral infection, followed by wastewater treatment plants and municipal solid waste landfills. Our QMRA approach in occupational settings is novel, and certain caveats should be considered. Nonetheless, we believe it is worthy of further discussions and investigations.

## 1. Introduction

Occupational biological risk assessment is particularly difficult because of the wide range of pathogenic agents, different types of exposure, the stochastic nature of infections, the presence of workers with different levels of susceptibility to risk, and the lack of epidemiological data that would permit the establishment of certain limits of exposure. Biological agents can pose serious risks in many work settings, mainly when they are transmitted through bioaerosols, defined as airborne particles with a biological origin [1].

Bioaerosols include bacteria, fungi, pollen, viruses, their fragments and byproducts (e.g., endotoxins and mycotoxins), and products or fragments from living beings, such as animal allergens [2]. Health effects that are caused by exposure to bioaerosols and can severely impact public health include infectious diseases, acute toxic effects, allergies, and cancer. The most widely studied and probably most important bioaerosol-associated health effects [3] are respiratory symptoms and impairment in lung function.

Despite recognizing the importance of bioaerosol exposure with regard to human health, the assessment of occupational biological risk is generally limited to evaluating “potential exposure”, without any quantitative estimate. This evaluation is based on the concept that biological risk is stochastic thus even a single microbial infectious particle can theoretically cause infection in an exposed worker. Nevertheless, the probability of this event depends on the infectivity of the pathogen, its concentration in the air, and exposure time. The risk assessment output is then usually expressed as a level (low, medium, high) and represented in risk matrices combining magnitude and probability scores [4].

When risk comes from environmental contamination, such as exposure to bioaerosols, microbiological monitoring can help define levels of risk and the sources and spread of biological agents [5]. Usually, this monitoring considers a generic contamination, measured as total bacterial count and total fungal count (TBC and TFC, respectively). Less frequently, and only in situations where faecal contamination occurs, an *Escherichia coli*-coliform count (ECC) is used. These indicators are not representative of all pathogens and cannot be correlated with the real risk, but rather only with general hygienic conditions [6].

In some cases, monitoring includes pathogens; however, owing to the lack of information on dose-response relationships and threshold limit values, risk management is based on the “precautionary principle”, and prescribes the adoption of preventive measures independent of the dose of the exposure. Occupational exposure limits (OELs) are rarely recommended in legislation, but several countries have proposed limits, mainly for total number of bacteria, gram-negative bacteria, bacterial endotoxin, and fungi [4,7,8]. 

Based on experience from biological risk assessments for drinking water and food [9,10,11,12], one possible way of estimating the risk from infectious agents is to use the Quantitative Microbial Risk Assessment (QMRA) methodology. This is a process of estimating the risk from exposure to pathogenic microorganisms (or a medium in which pathogens occur) by combining dose-response information for the infectious agent with information on the distribution of environmental contamination [13]. This methodology could also be useful for occupational risk assessment by allowing risk management to be planned on the basis of a defined acceptable risk and the simulation of prevention measures.

QMRA was derived from the chemical risk assessment paradigm that was set forth by the National Research Council (NRC) in 1983 [14,15], consisting of four basic stages: (*i*) hazard identification; (*ii*) exposure assessment; (*iii*) effect assessment (dose-response relationship); and (*iv*) risk characterization. In occupational settings, these stages should take into account the worker’s activity and identify the transmission chain, routes of exposure, and matrices involved. It is also essential to choose one index pathogen (or several) representative of the routes of exposure and infection and that is well known in terms of dose-response, resistance, pathogenicity, ease of detection and enumeration in environmental matrices. Although the measurement of pathogens in the air is more difficult than measurements in water or food, biomolecular methods represent an important technical progress for a rapid and easy detection of specific microorganisms in “difficult” matrices [16].

One possible innovative approach for risk assessment in occupational settings that are characterized by bioaerosol exposure is monitoring the air for a pathogen, which can be further used for QMRA. The choice of an index pathogen depends heavily on the sources of aerosol, environmental spread, resistance, and routes of exposure and infection. In many cases, the bioaerosol comes from water or dust contaminated by faecal or respiratory secretions, as in wastewater treatment [17], waste processing [18] or, more simply, from toilets [7].

Human adenoviruses (HAdVs) are the agents for numerous symptomatic and asymptomatic infections affecting the respiratory tract, the eyes, and the gastrointestinal tract. They can be excreted in the feces, urine, and respiratory secretions and transmitted via contact with the eyes, the fecal-oral route, or inhalation. HAdVs have a number of features that justify their use as index pathogens for air in occupational settings possibly contaminated by faecally-excreted pathogens. In fact, HAdVs have been suggested as virological markers for water quality because of their high concentrations in environmental waters and resistance to disinfection [19,20,21,22]. HAdVs can be pathogenic by either a respiratory or faecal-oral route of exposure, and the relationship between dose and infection in these types of exposures has been studied [13,23]. Moreover, methods of culture and biomolecular research and quantification of HAdVs are relatively simple and standardized.

For these reasons, we decided to use HAdVs as the reference pathogen for our study, the objective of which was to develop a preliminary QMRA model to assess the microbial risk associated with the inhalation of contaminated bioaerosols. This model was then applied to data on air contamination from different settings (e.g., wastewater treatment plants, solid waste landfills, toilets in offices and hospitals) and with various exposure times.

## 2. Materials and Methods

In previous studies, we quantified and reported HAdVs in the air in several settings [7,17]. In the present study, we report only information on the sampling sites and analytical methods, referring to previously published papers for more details [7,18].

### 2.1. Study Settings

The present study was performed using data obtained by monitoring bioaerosols in different occupational settings.

- Toilets in healthcare settings and offices. The airborne spreading of HAdV can occur through the aerosol and droplets produced by toilet flushing, contaminating the surrounding environment. The environmental monitoring of air and selected surfaces was carried out with five replicate sampling sessions, in three toilets in a hospital ward (i.e., one toilet for a four-bed patient room, one toilet for a two-bed patient room, and one toilet for healthcare personnel) and two toilets in an office building, for a total of 43 samples [7].
- Wastewater treatment plants (unpublished data). Aerosol produced by public sewage treatment plants may contain HAdVs that—because of their high stability under environmental conditions and likely transmission by the aerosol route—constitute a potential health hazard for plant workers and nearby residents. During a monitoring aimed to evidence the airborne contamination of 20 wastewater treatment plants, twenty-five samples were collected in areas that are at greatest risk of bioaerosol production: (*i*) entrance sewage as entry point of wastewater treatment plant; (*ii*) sludge treatment systems; (*iii*) biological oxidation tank; and (*iv*) side-entrance manhole.
- Solid waste landfill. The workers involved in the management of solid waste are at risk of exposure to bioaerosol which is a mixture of particles of biological origin or with biological effects such as bacteria, fungi, and viruses. To evaluate the presence of HAdV, sixteen samples were collected from eight sampling sites that were chosen based on their relevance to worker exposure [18]. Four of these sites were in the recycling paper area, and four were in an outside area.

### 2.2. Sampling Procedures and Virological Analysis

Air samples (1000 L of air in an indoor workplace and 3000 L in an outdoor area) were collected using an impactor sampler (Microflow Aquaria Srl, Milan, Italy) that was loaded with Rodac plates that contained tryptone soy agar (Oxoid, Milan, Italy). According to a previously-described protocol, the sampling agar was eluted in 15 mL of 3% beef extract (pH 9), and the supernatant was collected after mixing and centrifugation. The recovery efficiency of the elution/mixing/centrifugation method has been estimated to be on average 40%, ranging from 19% to 68%, for enteroviruses with cell cultures [24]. Viral DNA was extracted using the QIAamp Viral DNA Mini Kit (Qiagen, Hilden, Germany) starting from 200 μL of sample. The genomic concentration of positive samples was measured by real-time polymerase chain reaction (q-PCR) based on published protocols for HAdVs [25]. The samples were tested in triplicate and analyzed in 96-well optical plates using an ABI 7300 sequence detector system (Applied Biosystems, Foster City, CA, USA). To guarantee the quality of the assay-specific DNA positive control, a non-template control and a control for the presence of enzymatic inhibitors (uracil *n*-glycosylase) were used for each reaction. The results were expressed as the number of genomic copies per cubic meter (GC/m^3^).

### 2.3. Statistical Analysis and Simulation Tool

For each setting, the log (base 10)-transformed data on bioaerosol contamination were used to calculate means and standard deviations using GraphPad Prism 5.0 software (GraphPad Software, Inc., La Jolla, CA, USA). To calculate means and standard deviations for the experimental data by taking into account negative samples, we assigned a value of half of the detection limit of the analytical method (1 log GC/m^3^) [18,26].

The simulations for the static QMRA model were performed using Vensim (Ventana Systems, Inc., Harvard, MA, USA) [27], based on the metaphor of System Dynamics [28,29,30,31,32] for simulating the behavior of complex systems through models that consist of levels, flows, and other types of variables (constants and auxiliary variables).

### 2.4. QMRA Framework

A point-estimate QMRA model was elaborated according to the method suggested by a previous report [33] and articulated in four phases: (*i*) hazard identification; (*ii*) exposure assessment; (*iii*) effect assessment (dose-response relationship); and (*iv*) risk characterization.

#### 2.4.1. Hazard Identification

As indicated in the Introduction above, the hazard was considered to be an infection following the inhalation of HAdVs that are present in bioaerosols in different occupational settings. Given that HAdVs are largely widespread, this hazard could represent a general viral hazard that comes from bioaerosols.

#### 2.4.2. Exposure Assessment

The calculation of the inhaled dose should take into account various factors: inhalation rate (r_in_), exposure time (t_exp_), and concentration of HAdVs in the air (expressed as GC/m^3^).

The inhalation rate varies with activity level, age, weight, sex, and general physical condition [34]. In our study, we used the value based on 16 h light activity for adult males.

Although quantitative polymerase chain reaction has become a useful method for virus detection [16], its recovery efficiency is generally less than 100%. It can detect virus-specific nucleic acids, but it does not allow conclusions to be drawn regarding virus infectivity [35,36]. For this reason, in the present model, we considered the estimated virus concentration based on recovery efficiency (r_eff_). We used a conversion factor (f_conv_) that allowed us to obtain infectious viral particle (PFU) values by taking into account the proportion of genomic copies (GCs) that corresponds to the infectious virus [37]. Based on these considerations, we derived the following expression:

dose = (HAdV)/r_eff_ × r_in_ × t_exp_ × f_conv_ (1)

#### 2.4.3. Dose-Response Relationship

An essential part of QMRA is a suitable dose-response model for estimating the probability of infection that is caused by exposure to an infectious agent. The only information about the dose-response relationship for HAdV available at present from the literature is the one derived from clinical trials [23,38] and further elaborated by Haas [39]. These studies investigated the dose-response relationship for respiratory effects in volunteers (healthy adult males) exposed to aerosol that contained Human Adenovirus type 4. The mathematical function (see Equation (2) below) that was formulated [13] provided the best fit for the inhalation of adenovirus-containing aerosols in humans is a single-parameter exponential function.

#### 2.4.4. Risk Characterization

Risk characterization is the final phase of risk assessment, consisting of three phases: hazard identification, dose-response assessment, and exposure assessment. This phase determines the risk of infection, computed using an exponential dose-response model [13]:

P_r_ = 1 − exp (−r × dose) (2)

where the dose is expressed by Equation (1). The model input parameters were based on the scientific literature and their mean values were used as constants in the model (Table 1).

## 3. Results

**Figure 1:**
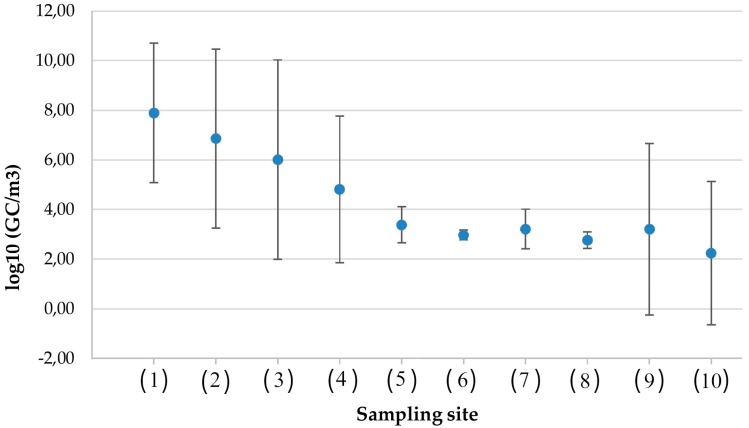
Concentrations of HAdV in different occupational settings. Sampling sites (average value μ and intervals (μ − σ, μ + σ), σ = standard deviation (SD)). Toilets: (1) four-bed patient room (7.90 GC/m^3^, SD = 2.81); (2) two-bed patient room (6.86 GC/m^3^, SD = 3.61); (3) healthcare personnel (6.02 GC/m^3^, SD = 4.02); (4) office building (4.81 GC/m^3^, SD = 2.96); wastewater treatment plants: (5) entrance sewage (3.39 GC/m^3^, SD = 0.73); (6) sludge treatment (2.97 GC/m^3^, SD = 0.20); (7) biological oxidation tank (3.21 GC/m^3^, SD = 0.80); (8) side-entrance manhole (2.77 GC/m^3^, SD = 0.33); solid waste landfill: (9) paper recycling (3.20 GC/m^3^, SD = 3.46); (10) outside area (2.24 GC/m^3^, SD = 2.89).

Virological monitoring detected HAdVs in all of the sampled settings (Figure 1), thus confirming their widespread presence. Nevertheless, the average concentrations of HAdV differed, ranging from 2 log_10_ GC/m^3^ in the area outside the landfill during the winter to 8 log_10_ GC/m^3^ in hospital ward toilets. The indoor environments had the highest concentrations of HAdVs. For this reason, these areas must be considered to be at a greater risk for exposure and infection.

Figure 1 shows a graphic representation of descriptive statistics for the HAdV concentrations in different occupational settings.

**Figure 2:**
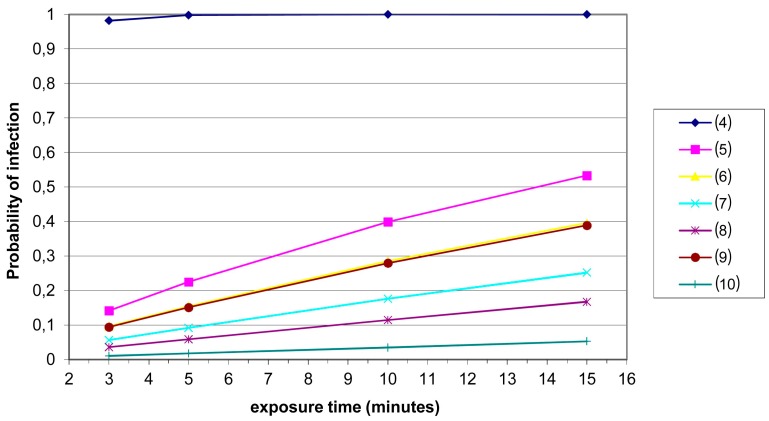
Probability of infection as a function of exposure time in different occupational settings. Sampling sites (average values μ): toilets: (4) office building (4.81 GC/m^3^); wastewater treatment plants: (5) entrance sewage (3.39 GC/m^3^); (6) sludge treatment (2.97 GC/m^3^); (7) biological oxidation tank (3.21 GC/m^3^); (8) side-entrance manhole (2.77 GC/m^3^); solid waste landfill: (9) paper recycling (3.20 GC/m^3^); (10) outside area (2.24 GC/m^3^).

For each occupational setting, we verified a trend of probability of infection as a function of exposure time (in minutes). Such times were chosen from the set {3,5,10,15}. Temporal trends for hospital toilets are not shown in Figure 2 because we obtained a probability of infection of 1 after 1 min for these settings.

## 4. Discussion

Directive 2000/54/EC of the European Parliament and Council of 18 September 2000 [38] on the protection of workers from risks related to exposure to biological agents at the workplace deals mainly with the risk of infectious agents and gives guidance on health surveillance and containment levels. However, exposure limits are not given for either infectious or non-infectious biological agents, thus implying that simply the potential presence of a pathogen in the air should require the use of respiratory personal protection equipment (RPPE), regardless of exposure time. This practice can be difficult to accomplish in many occupational settings (e.g., outdoor plants for wastewater treatment and landfills) where air contamination differs between areas. Additionally, wearing RPPE can be very uncomfortable. In many cases, workers may not comply with the regulations. The application of QMRA frameworks and models could be useful to give operational guidance for risk management and could lead to a better understanding of health risks from exposure in workplaces.

The use of QMRA to estimate risk associated with drinking water, food, bioaerosols, and fomites was proposed in several studies as a valuable tool to evaluate regulatory standards, bridge information gaps, and assist risk managers in making informed decisions. For example, QMRA has been used [39] to assess the risk associated with enteric and skin pathogens via exposure to contaminated fomites and evaluate risk reductions that are needed to achieve a safety goal of 1 × 10^−6^. The use of such models to predict potentially critical human exposure to *Legionella* has been examined in various studies [40,41,42] where, unlike our study, the concentration in air was estimated based on levels in the water. Moreover, in a study that was performed in southern Italy [43], the QMRA approach was used to assess the risk from a sewage treatment plant for a nearby population. Rotavirus, *Campylobacter*, and *Cryptosporidium* were selected as index pathogens. Similarly, in another study, QMRA was used to quantify the associated public health risk relative to the distance downwind from the manure application area [44]. Some of the settings we considered can also be contaminated by microbial metabolites (for instance endotoxins and gliotoxin) that could increase the viral infectivity. This was the case of solid waste processing facilities, where endotoxins were sometimes found to be at high levels [18,45]. The synergistic effect of these substances could also be taken into account in a QMRA, if they could be quantifiable, but at the present time these data are unavailable.

In the present study, we applied QMRA methodology to workplace settings in order to determine the risk of infection that is caused by inhalation exposure to HAdV, which was chosen as a reference pathogen because of its wide dispersion, resistance, and infectivity [46]. Our data confirmed the wide diffusion of this virus and also found considerable differences between indoor and outdoor settings. Our findings reveal an opportunity to reduce indoor contamination with adequate ventilation systems.

The simulations that employed empirical data showed that going to an office toilet for 3 min may be associated with a higher HAdV infection risk compared with working for 15 min at the entrance of a wastewater treatment plant. These results suggest the implausible need to wear RPPE to go to the toilet, more than when working in wastewater treatment plant areas. Such a finding could derive from the index pathogen that was chosen (HAdV). All adenovirus genomic copies were indeed included in the assessment to yield the maximal estimate of risk, although only a sub-portion of the 51 adenovirus serotypes are known to cause respiratory illnesses [47]. Of the 51 known adenovirus serotypes, only one-third are associated with human disease, and infections by other serotypes are asymptomatic [48]. The dose-response of adenovirus serotype 4 should not be strictly applied to all adenoviruses. Moreover, the presence of other pathogenic agents (e.g., *E. coli*, *Salmonella*, reovirus, enterovirus, norovirus, hepatitis A virus, and rotavirus) could be associated with a higher risk of infection in settings where aerosols are contaminated by sewage [17,49,50,51,52]. Finally, although the objective of the study was limited to assessing the infectious risk due to viral inhalation in occupational settings, we are aware that other contaminated matrices, such as surfaces, can be related to possible exposures. Nevertheless, specific data on dose-response relationships for the exposure to contaminated surfaces are unavailable, owing to the multiple ways of infection related to them: not only skin contact, but also hands, food, and tool contamination. In order to take into consideration multiple pathogens and multiple exposures, the QMRA model should be more complex and should use dose-response data specific for each agent and route of transmission. 

## 5. Conclusions 

In conclusion, the QMRA approach—applicable to occupational settings where workers’ exposure depends on environmental contamination—can be a useful tool in order to establish exposure limits in terms of pathogen concentration and/or exposure times, to identify the relative importance of different risk management options (e.g., use of RPPE) and in general to predict, simulate, and optimize preventive measures. Nevertheless, this methodology is new in this area and should be used with caution. For a further study on a specific setting, the proposed methodology requires further refinements that include both the use of probability distributions instead of point estimates (in order to take into account the uncertainties and the variabilities on some of the parameters with Monte Carlo techniques) and the gathering of more data of good quality on dose-response relationships, efficiency of the method, and inhalation rates. Our work is just the beginning of a wider study on occupational risk assessment based on QMRA that will be aimed to face the different transmission vehicles and routes of exposure. However, this is the first tentative approach of using QMRA for occupational biological risk assessment, and its future developments could be very useful in risk management.

## Figures and Tables

**Table 1:** Input parameters used in the exponential dose-response model.

Parameter	Value	Reference
Recovery efficiency: r_eff_	40%	[18]
Single parameter-model for HAdV: r	0.4172	[33] **^1^**
Conversion factor: f_conv_	10^−3^ PFU/GC	[37] **^2^**
Average inhalation rate: r_in_	1.2 m^3^/h	[34] **^3^**

**^1^** The reported value is the best-fit parameter of the model [33]; **^2^** The only value for Human Adenovirus (HAdV) infectivity available in the literature and derived from the studies of Couch, et al. on HAdV type 4 [23]; **^3^** The inhalation rate varies with activity level, age, weight, sex, and general physical condition [34]. In our study, the chosen value was based on 16 h light activity for adults. GC: genomic copy; PFU: infectious viral particle.

## Abbreviations

The following abbreviations are used in this manuscript:

QMRA	Quantitative Microbial Risk Assessment
HAdV	Human Adenovirus
